# Prediction of plant food allergens using protein embeddings

**DOI:** 10.1093/bioadv/vbag114

**Published:** 2026-04-26

**Authors:** Martín Méndez, Francisco Javier Moreno, Florencio Pazos

**Affiliations:** Computational Systems Biology Group, National Centre for Biotechnology (CNB-CSIC), Madrid 28049, Spains; Instituto de Investigación en Ciencias de la Alimentación (CIAL), CSIC-UAM, CEI (UAM+CSIC), Madrid 28049, Spain; Computational Systems Biology Group, National Centre for Biotechnology (CNB-CSIC), Madrid 28049, Spains

## Abstract

**Motivation:**

The current trend to incorporate new protein sources to the diet, mostly from plants, requires a safety assessment of these polypeptides when used as food. Part of that safety evaluation involves assessing the potential allergenicity of the proteins. Such assessment typically follows a hierarchy of *in silico*, *in vitro* and *in vivo* assays. The computational prediction of protein allergenicity is the first step in the process and several predictors are available for this task. Due to the complexity and heterogeneity of the allergy phenomenon, the large differences between allergens in their mechanisms of exposure and action, and the role of their inherent biological and physicochemical properties, predictors focused on particular organisms or subsets of allergens are in principle better than general predictors.

**Results:**

In this work we present a predictor of protein allergenicity focused on plant food allergens. The predictor, trained on known plant food allergens and bona-fide plant non-allergens, represents proteins by their sequence embeddings, a deep-learning based representation known to capture important features of a protein’s function and properties. The predictor outperforms the typical prediction based on sequence similarity to other allergens, especially in terms of sensitivity. In spite of being trained on plant food allergens, the predictor also performs well when applied to allergens from other sources.

**Availability and implementation:**

The system is available as an open-source package at Github: https://github.com/Martinmendezlopez/ALLERGEN-PREDICTOR-FROM-ProtTrans/

## 1 Introduction

Demands for healthier and more sustainable food systems have driven rapid advances in biotechnology, the formulation of increasingly complex foods, and an intensified search for alternative protein sources. In particular, the rising preference for non–animal-based proteins, including plant-based meat alternatives and novel plant or microbial ingredients, promises environmental and public-health benefits but also introduces the possibility of exposing both allergic and non-allergic consumers to previously unseen food proteins (Kopko *et al.* 2022, Jafarzadeh *et al.* 2024). Consequently, the safety assessment of such products must now address not only traditional hazards but also the allergenic potential of many new protein sequences.

Indeed, food allergies are a growing public health problem, affecting millions of people worldwide, including up to 8% of children (Oyoshi and Chinthrajah 2020). As there is no definitive cure for this life-long condition, the detection of potential allergens and the subsequent food labelling is crucial.

Due to the time, cost and expertise required for experimental approaches to evaluate a protein’s allergenic features (Marzano *et al.* 2020), *in silico* prediction is the first step in allergenicity evaluation workflows. Predicting allergenicity is a major challenge in this context because an allergic reaction to a protein depends on a complex interplay between the individual’s immune system, the intrinsic biological and physicochemical properties of the protein, and a range of environmental and lifestyle co-factors (e.g. microbiome composition, pollutants, diet, and adjuvant exposures) (Mullins *et al.* 2022, Falcon and Caoili 2023, Caminero *et al.* 2025). Clinically relevant outcomes range from mild oral symptoms to life-threatening anaphylaxis, and population prevalence varies by geography, age and exposure patterns; thus, even low-frequency sensitization events can have important public-health consequences when widely consumed novel proteins are involved. Current strategies and tools used for risk assessment and management of food allergies are still evolving and face important limitations (Mazzucchelli *et al.* 2018, Remington *et al.* 2018, Food allergy is everyone’s business 2021). Allergenicity prediction therefore represents a key, unresolved component of the safety evaluation of foods produced through biotechnology or containing novel proteins (Fernandez *et al.* 2021, 2024).

Allergenicity prediction encompasses two distinct objectives: (i) assessing the potential for IgE‑mediated cross‑reactivity in individuals already sensitised to related allergens, and (ii) evaluating whether a protein may induce a *de novo* sensitisation in previously non‑allergic individuals. Current *in silico* approaches for assessing potential cross‑reactivity are comparatively more developed than those aimed at predicting *de novo* sensitisation, but they still rely heavily on sequence similarity and have known limitations when dealing with divergent or novel proteins. This highlights the need for improved computational tools, such as embedding‑based models, that capture broader molecular features beyond explicit homology (Mullins *et al.* 2022, Casacuberta *et al.* 2025). Likewise, the prediction of *de novo* sensitisation remains highly uncertain because the mechanisms leading to the development of food allergy are not yet fully elucidated (Mills *et al.* 2024, Laganaro *et al.* 2025).

The current *in silico* standard for allergenicity screening is based on amino-acid sequence similarity to known allergens, operationalized historically by the WHO/FAO criterion of ≥35% sequence identity across a sliding window of at least 80 amino acids (Evaluation of allergenicity of genetically modified foods: report of a Joint FAO/WHO Expert Consultation on Allergenicity of Foods Derived from Biotechnology, 22–25 January 2001 n.d). This rule-based criterion was incorporated into Codex Alimentarius guidance (World Health Organization, Food and Agriculture Organization of the United Nations 2009) as a conservative filter to flag potential cross-reactive proteins for follow-up testing. However, several authors have noted important limitations when the criterion is applied broadly (e.g. to large-scale protein screens or to predicted open reading frames) because it yields many false positives and has limited discriminative power for diverse protein families (Ladics *et al.* 2007, Harper *et al*. 2012, Young *et al.* 2012, Abdelmoteleb *et al.* 2021, Herman *et al.* 2021). These shortcomings underscore the need for more informative *in silico* methods that can overcome the sensitivity–specificity trade-off of current sequence-similarity criteria and provide mechanistic interpretability to support weight-of-evidence decision-making (Casacuberta *et al.* 2025).

In this context, a number of methods for the *in silico* prediction of protein allergenicity using approaches beyond plain sequence similarity were developed (i.e Dimitrov *et al.* 2014, Sharma *et al.* 2021, Nguyen *et al.* 2022, Zhang and Liu 2024, Kemmler *et al.* 2025, Sarlakifar *et al*. 2025). Many of these methods are based on machine learning techniques: they are trained with examples of allergenic and non-allergenic polypeptides. In most cases, they are general predictors, trained to predict allergenicity irrespective of the type of allergen, mechanism or source, instead of focused on particular subsets of allergens. Due to the scarcity of bonna-fide sets of non-allergenic proteins, for building the large sets of negatives they require for training (the set of positives is large due to their generality commented earlier) these methods have to make assumptions, such as including human proteins or other proteins suspected to be non-allergenic.

There prediction tools require some sort of representation of the protein that can be handled by the machine learning method, including k-mers, amino-acids descriptors/statistics and QSAR-based parameters. A protein vectorial representation that is being increasingly used for many different purposes is that of “protein embeddings” (Simon *et al*. 2024). These embeddings are latent codifications of proteins and proteins residues obtained from the internal layers of pre-trained deep neural networks. In spite of the difficulty in translating these vectors and their components to interpretable features of the coded proteins, they have been shown to be tremendously useful in classification and predictive tasks, as these representations capture extensive information on a protein’s structure and function (Rives *et al.* 2021).

In this work, we present a predictor of protein allergenicity specifically focused on plant food proteins. We evaluate the classifier on a curated dataset of experimentally validated plant food allergens and *bona-fide* plant non-allergens. We show that the embedding-based predictor outperforms classical similarity-based method, especially in specificity. In spite of its focus on plant foods, it generalizes well to other allergen types, and is released as an open-source package to support reproducibility and community use.

## 2 Methods

To assemble the dataset used for training and testing the predictor we started with an allergen collection previously compiled by Novoa *et al.* (2024) combining the two main allergen databases available. That set of allergens comprises proteins for which there are experimental evidences on their allergenic character, either in vitro or in vivo. From that, we took the allergens of “plant food” origin (462 proteins), as our set of positives. As a set of negatives, we used the 178 proteins compiled by Krutz *et al.* (2019), which were abundant proteins from commonly consumed plant foods (i.e. corn, potato, rice, tomato, spinach, wheat) for which there is no evidence of allergenic activity, despite high levels of human exposure. These authors carefully curated this set of *bona-fide* plant food non-allergens by selecting plant food proteins based on their high abundance, predicted low similarity to known allergens, and structural features indicating low allergenic potential.

**Figure 1:**
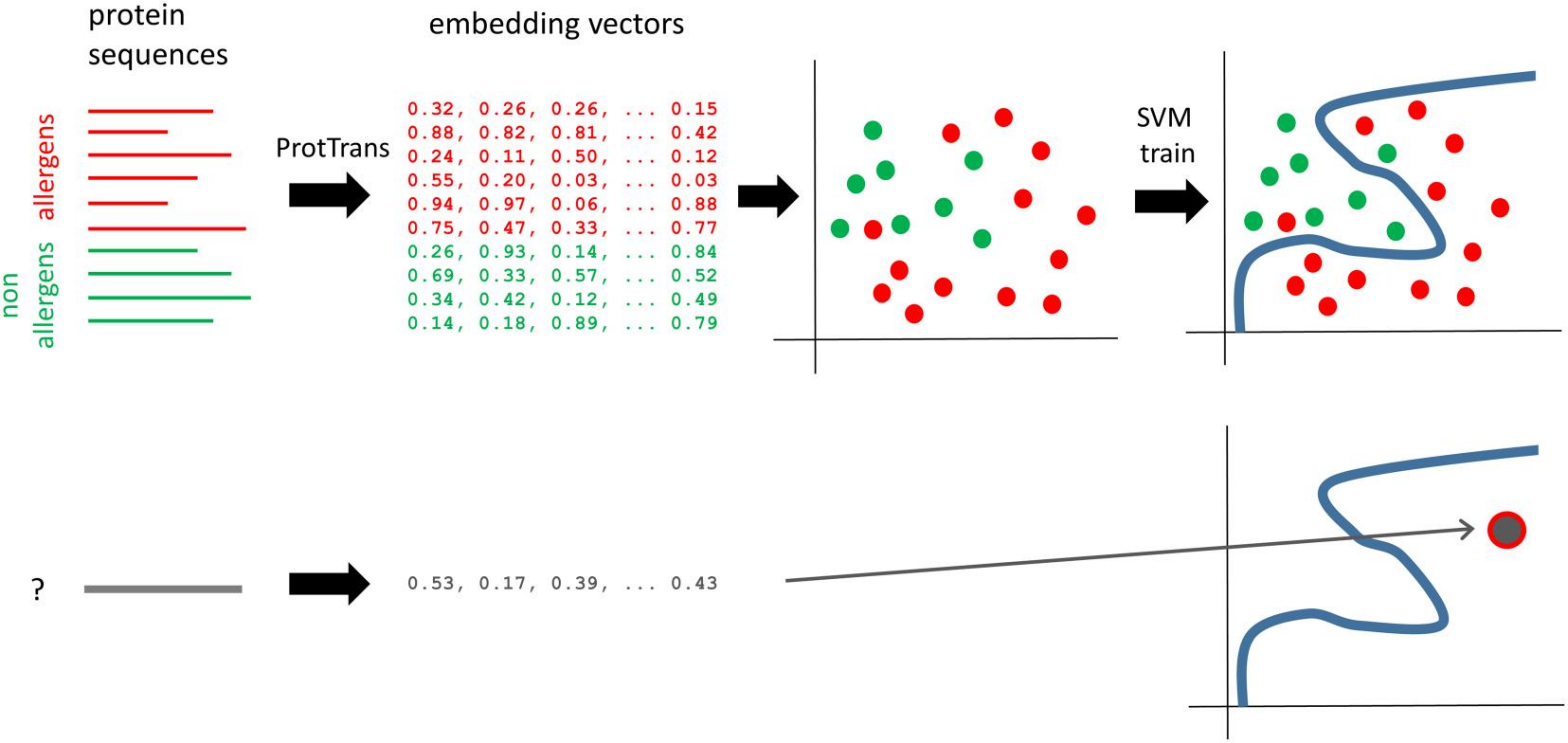
Schema of the binary classifier. The upper part of the figure illustrates the training of the predictor. The sets of plant food allergens (red) and plant non-allergens (green) are transformed to their corresponding embeddings with ProtTrans. SVM is used to find an optimal hypersurface to separate these two sets of embedding vectors (blue curve). Once that surface is defined, a new sequence can be classified by looking at which part of the surface its embedding vector falls (“allergen” in this illustration).

From the amino-acid sequences of these 640 proteins we obtained the embedding representations generated by the ProtT5 pre-trained model implemented in ProtTrans (Elnaggar *et al.* 2022). We generated per-protein embeddings (“—per_protein 1” option of the command line). These particular embeddings represent a protein’s sequence as a vector of 1024 components, irrespective of its length (Fig. 1). We applied Principal Component Analysis (PCA) for projecting these vectors into a space of lower dimensionality so as to visualize their clustering structure.

In order to generate a binary predictor, we use a Support Vector Machine (SVM) approach. This method tries to find the hyper-surface that best separates two sets of vectors (Fig. 1). Once determined using a training set of vectors, such surface can be used to assign new (unseen) vectors to the classes depending on which side they fall. Additionally, the distance to that surface can be used as a measure of confidence in the prediction, as vectors closer to that border delimiting the two classes are expected to be less confidently classified.

We employed a *k*-fold approach to train and test with the same dataset at the same time that all items are tested and in experiments where they are not part of the training sets. For that we divided randomly the dataset in *k* parts, used *k*-1 of them for training, and tested in the other, cycling the procedure k times in order to cover all parts. In our case we used *k* = 10.

As kernel for the SVM we used the “radial basis function,” with an “error and penalty term” (C) of 100, and the gamma parameter set to “scale.”

With the *k*-fold strategy we obtain a prediction for each protein in our dataset, generated by a predictor that did not see it during training. This prediction can be contrasted against the known allergenicity for that protein, and hence labelled as right or wrong. We used the following metrics to evaluate the performance of these predictions:


$$\begin{array}{l} \text{Accuracy}=\left(\text{TP}+\text{TN}\right)/\left(\text{TP}+\text{TN}+\text{FP}+\text{FN}\right)\\ \text{Sensitivity}=\text{TP}/\left(\text{TP}+\text{FN}\right)\\ \text{Specificity}=\text{TN}/\left(\text{TN}+\text{FP}\right)\\ \text{Precision}=\text{TP}/\left(\text{TP}+\text{FP}\right)\\ \text{Balanced}\;\text{accuracy}=\left(\text{Sensitivity}+\text{Specificity}\right)/\;2 \end{array}$$

Where TP = “true positives” (allergens correctly predicted as such), FP = “false positives” (non- allergens incorrectly predicted as allergens), TN = “true negatives” (non-allergens predicted as such) and FN = “false negatives” (allergens incorrectly predicted as non-allergens)

To further evaluate the discriminatory capacity of the predictor we applied “Receiver Operator Curve” (ROC) analysis: the dataset is sorted by the prediction score and sensitivities/specificities are calculated for different score thresholds, generating a plot of sensitivity vs. 1-specificity. In this plot, a random predictor, uniformly placing positives and negatives irrespective of the score, would be represented as a diagonal, while a predictor placing positives at high scores and the other way around would be represented by a curve close to the top-left corner, the closer the better the discriminatory capacity. The “Area Under the Curve” (AUC) parameter can be used to quantify that closeness, as the random predictor (diagonal) would render an AUC value of 0.5, while this figure for a perfect predictor would be 1.0. We also generated precision/recall curves from the same ranked list.

Once the method was evaluated with the *k*-fold approach, we generated a final predictor trained with the whole dataset.

This final predictor was applied to two additional external datasets available in the literature containing allergens and (inferred) non-allergens from different sources: a dataset of 218 allergens and 212 likely non-allergens compiled by Maurer-Stroh *et al.* (2019), and another with 2003 allergens and 2015 potential non-allergens by Sharma *et al.* (2021). The final predictor was also applied to all allergens compiled by Novoa *et al.* (2024), excluding the 462 plant food allergens used in the training (1622 allergens). Note that this last dataset contains only positives (allergenic proteins) and it does not include negatives.

To compare with the prediction based on a standard sequence search, we used the BLAST program (Camacho *et al.* 2009). For each allergen and non-allergen in the test set, the most similar sequence is detected in two databases: one containing all allergens compiled by Novoa *et al.* (2024) (2084 sequences), and the other containing the subset of plant allergens along with *bona fide* non-allergens (640 sequences) used previously for training the SVM classifier. As the most similar sequence we take the BLAST hit (different from the sequence itself) with the highest sequence identity (as long as it exceeds 35%) and an *e*-value equal to or less than 0.01. If these conditions are not met, we consider that there is no similar sequence in the corresponding database. If the test sequence is an allergen, we consider a correct prediction when a similar sequence exists (with the criteria explained above) and it is also an allergen. For a non-allergen, the prediction is considered correct if there is no similar sequence or if that is from another non-allergen. This protocol tries to simulate a real-world scenario in which allergenicity predictions would be carried out searching the query sequences against databases of known allergens.

## 3 Results

**Figure 2:**
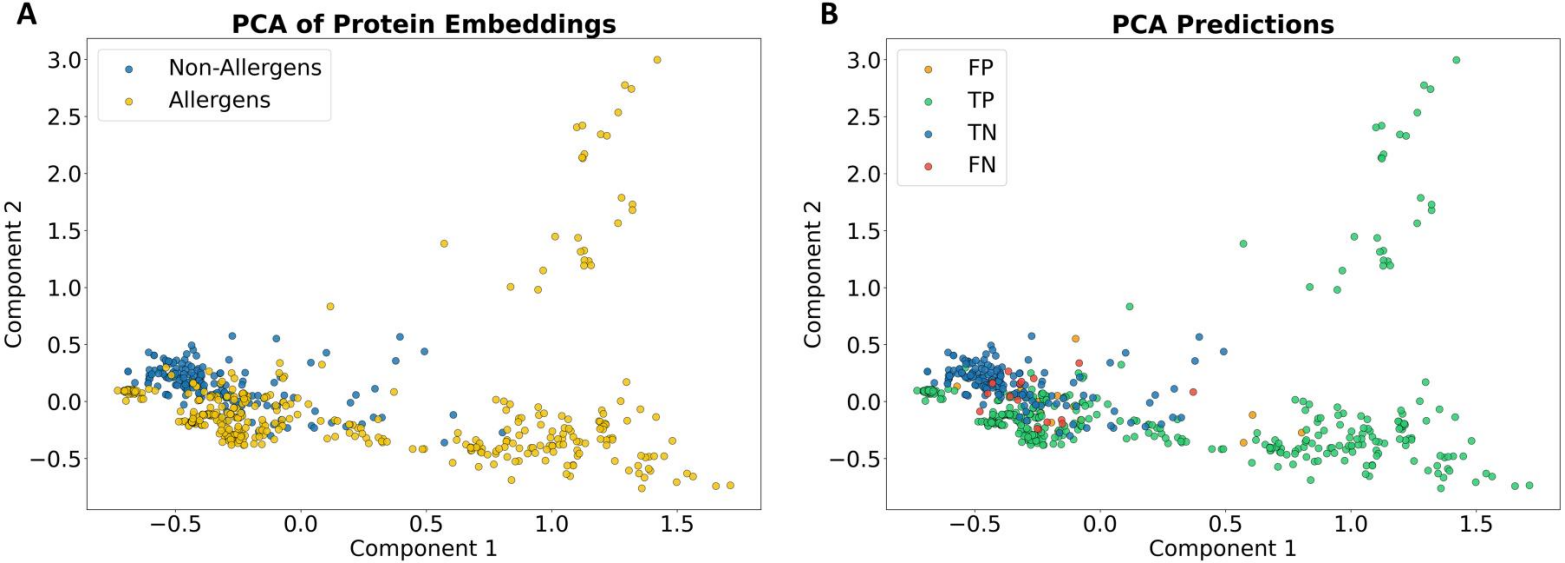
PCA visualization of the protein embedding vectors. The two principal axis of the reduced space are shown. (A) proteins are colored by their allergenicity: blue (non-allergens) and orange (allergens). (B) proteins colored by the prediction outcome: FP, false positives (non-allergens predicted as allergens); TP, true positives (allergens predicted as allergens); TN, true negatives (non-allergens predicted as non-allergens) and FN, false negatives (allergens predicted as non-allergens).

The vector embeddings of allergens and non-allergens show a certain separation, as shown by the PCA analysis (Fig. 2A), supporting the development of a binary classifier.

After obtaining predictions for all proteins with the k-fold procedure described in Section 2, the same PCA plot can be generated coloring by the prediction outcome, instead of the original class (Fig. 2B). It can be seen that most cases are correctly predicted (true positives and negatives), while the relatively few wrong predictions (false positives and negatives) are close to the boundaries of the clusters, as expected.

Table 1 shows the performance metrics of the binary predictor and those of the equivalent sequence similarity-based approach described in Section 2, using sequence searches against the plant allergens, as well as against the whole dataset, which includes allergens from other sources. The sequence search against the plant dataset represents a fairer comparison with our approach, trained with plant sequences only, while searching against all allergens represents a more real-world scenario. It can be seen that both approaches show good performances predicting allergens. Nevertheless, the SVM classifier is globally better, with an overall accuracy of 95% cases correctly classified. The difference in sensitivity is even higher, while the specificity is similar to that of a BLAST search or even slightly lower when the database of plant-only allergens is used. A higher sensitivity means that the predictor tends to generate less false negatives (allergens incorrectly predicted as non-allergens), while the slightly higher specificity of BLAST when searching against the plant database means that this program in that scenario tends to produce less false positives (non-allergens incorrectly predicted as allergens).

**Table 1:** Performance metrics for the embedding-based (SVM) predictor and for the equivalent strategy based on sequence similarity.

	Accuracy	Sensitivity	Specificity	Balanced accuracy	Precision
SVM predictor (plant DB)	0.95	0.96	0.94	0.95	0.98
BLAST (plant DB)	0.91	0.90	0.95	0.92	0.98
BLAST (whole allergen DB)	0.93	0.92	0.94	0.93	0.98

**Figure 3:**
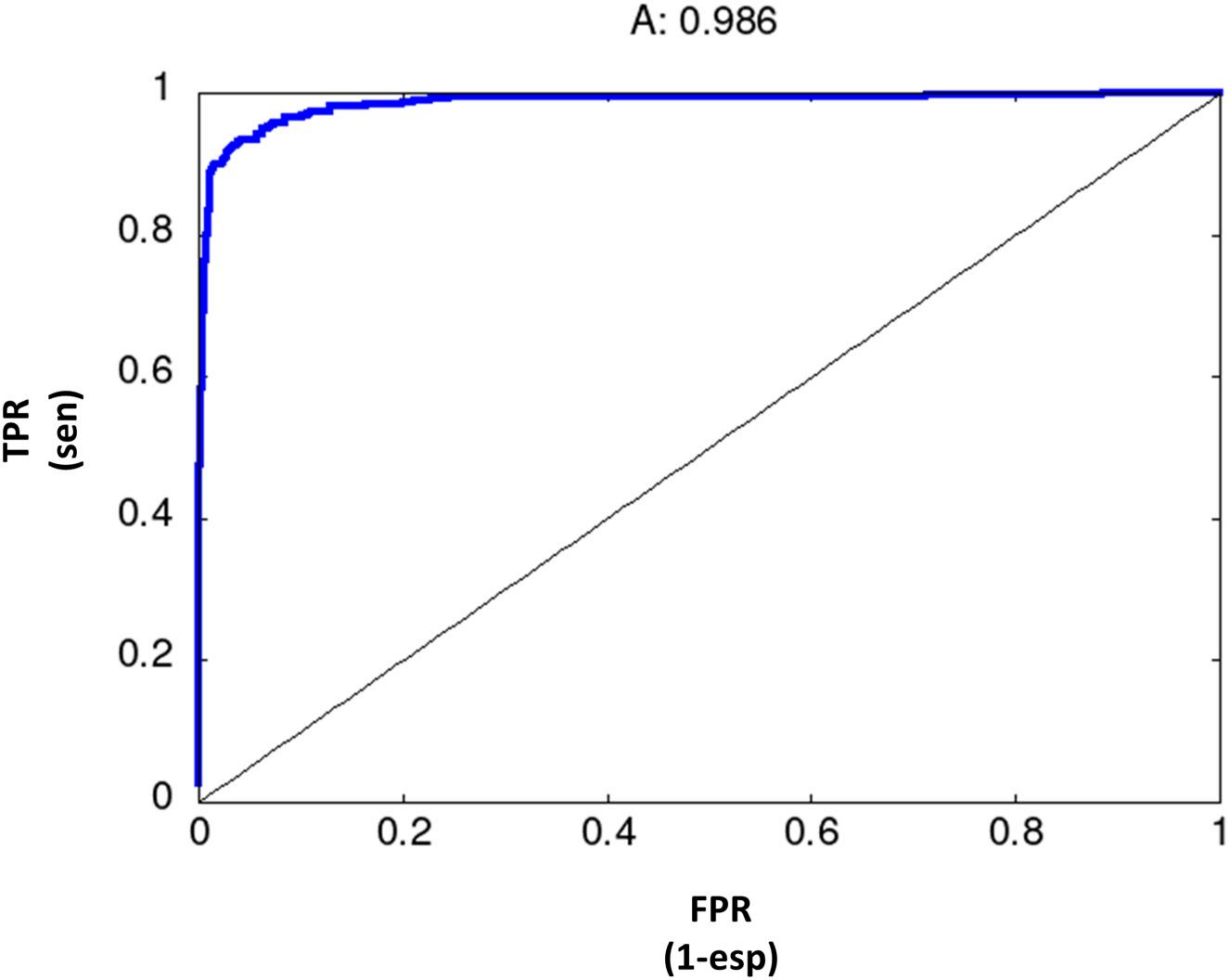
Receiver Operator Curve (ROC) plot illustrating the discriminative power of the method in the plant food allergens/non-allergens dataset.

The ROC plot representing the discriminative capacity of the method along the ranked list of scores is shown in Fig. 3. The curve, close to the top-left corner, indicates a very good discrimination, what is quantified by an AUC of 0.986 (in a 0.5 to 1.0 scale). The corresponding precision/recall curve is available in the Supplementary Material.

**Figure 4:**
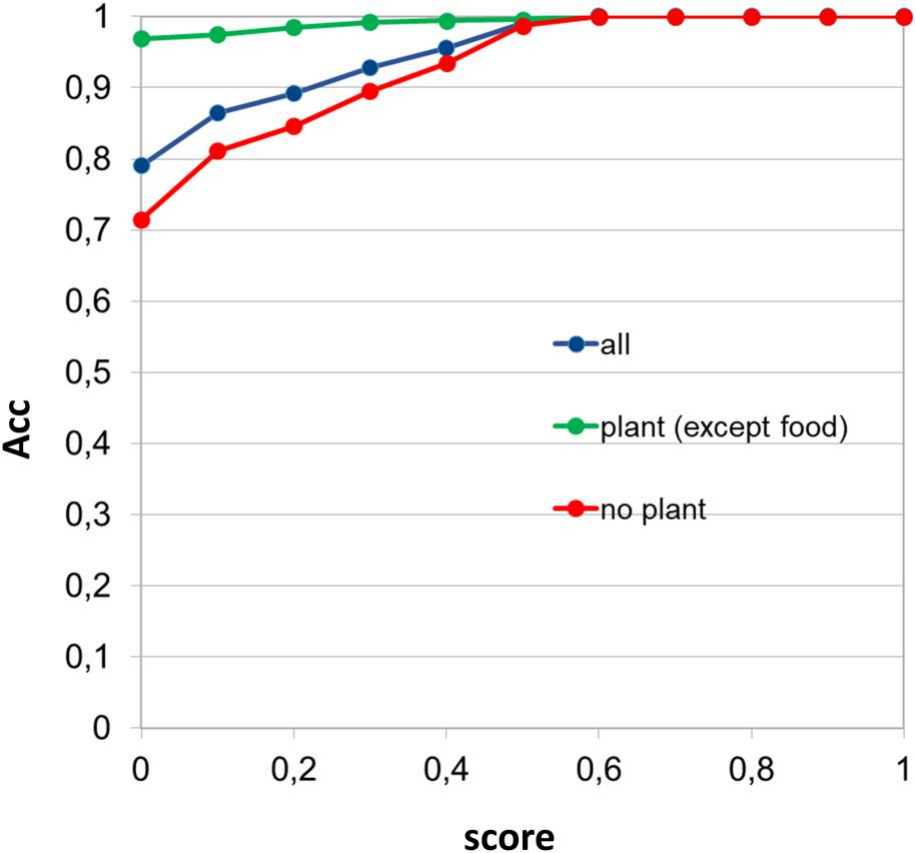
Results for the dataset including allergens from other sources. Proportion of allergens recovered (*Y* axis) at each score threshold (*X* axis). The score goes from 0.0 (good) to 1.0 (bad). The results for all allergens, those from plant origin but not annotated as “food allergen,” and those of non-plant origin are shown with different colors.

The results of the application of the final predictor to all allergens compiled by Novoa *et al.* (2024) (except those used for training the method –plant foods-) are shown in Fig. 4. This list of 1622 allergens still contains some allergens of plant origin but not classified as “food,” such as those from pollen. Another important point is that this list contains only “positives” (proteins annotated as allergens), and hence we can only evaluate the fraction of them detected at a given score threshold. The score of the method goes from 0.0 (very good), to 1.0 (bad). It can be seen that at score ∼0.5 all allergens are recovered (i.e. there are no real allergens of any type with scores worse than 0.5). Moreover, 80% of the allergens have the highest possible score (0.0). If we evaluate separately the allergens of plant (non-food) origin and those from other sources, those from plants show a much higher performance, expected as the predictor was trained with plant proteins.

**Figure 5:**
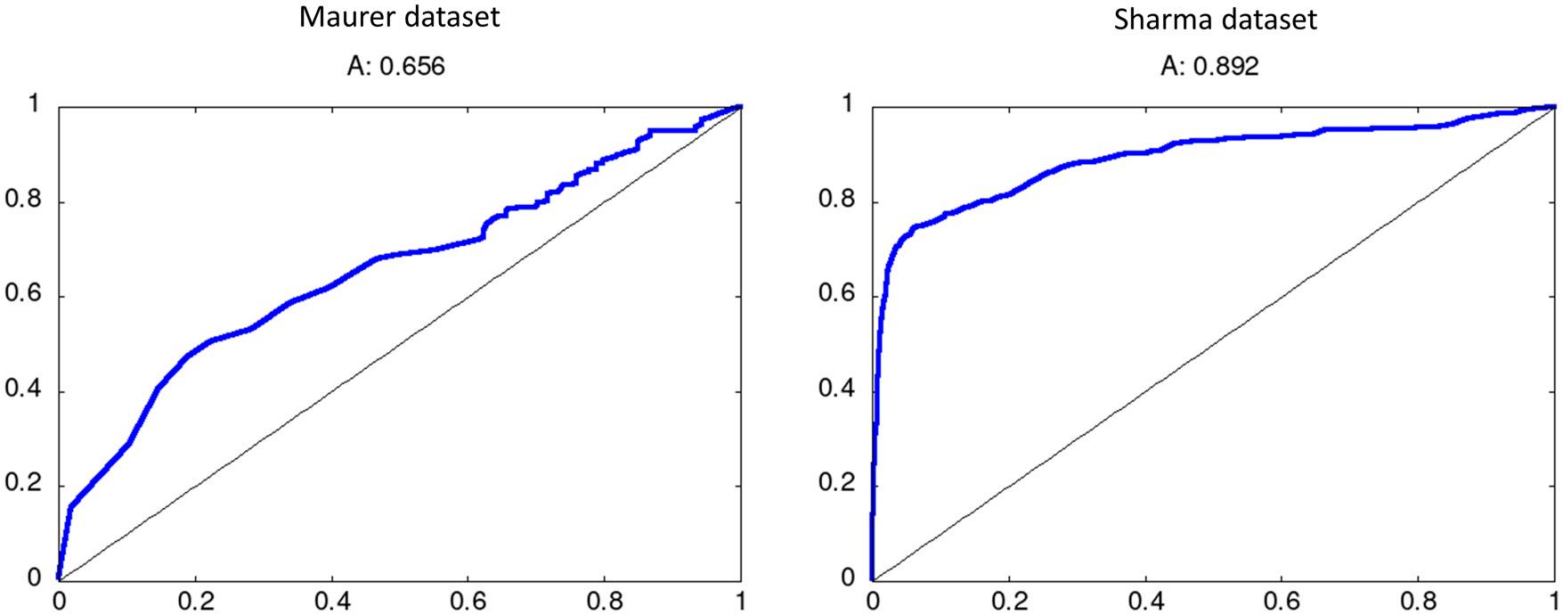
Results for two external datasets. ROC plots and AUC values for Maurer-Stroh *et al*. and Sharma *et al*. datasets.

Regarding the two datasets of allergens and non-allergens from different sources compiled by other authors, Fig. 5 shows the ROC plots evaluating the performance of the classifier for them. The performance is good in both cases (AUC values of 0.656 and 0.892 respectively), and particularly high for Sharma’s dataset, which contains a large number of proteins (4018). The corresponding precision/recall curves are available in the Supplementary Material.

## 4 Discussion

Predicting *in silico* allergenic features of proteins, as a first step in their safety assessment, is of crucial importance considering the current trend to incorporate new species as food sources. Most of the species that are being included (or are planned to be) as food sources are of plant origin. Hence, in this context an allergenicity predictor focused on plant proteins is desirable. Allergenicity is a very complex and heterogeneous phenomenon, with different allergens eliciting it via different mechanisms. Consequently, specific predictors are expected to be better than those more general. Another point in favor of developing a predictor focused on plant proteins is the availability of a curated set of “negatives” (*bona-fide* non-allergenic proteins), whereas existing general predictors assemble their negative sets based on different assumptions about (potential) non-allergens.

Our embedding‑based predictor is trained exclusively on proteins with experimentally demonstrated allergenicity and *bona‑fide* non‑allergenic plant proteins. Therefore, the model learns patterns associated with known allergenic proteins and can be interpreted as predicting the likelihood of cross‑reactivity. Importantly, the method cannot predict *de novo* sensitisation, as this would require modelling immunological mechanisms that are not currently understood well enough to *support in silico* prediction (Mills *et al.* 2024). Thus, despite focusing on cross‑reactivity, our method contributes to the practical needs of risk assessment for novel proteins, where improved sensitivity and more informative sequence‑derived representations can provide actionable early‑tier screening. By leveraging protein embeddings, our predictor extends the scope of current similarity‑based screens and improves the detection of potential allergenic signals in cases where classical homology methods perform suboptimally.

In spite of being focused on plant proteins, our predictor shows good performance on other types of allergens, and hence it can also be used as a general predictor, complementing other tools available for the same goal. The performance is also good in external datasets compiled by other authors, which also include non-plant allergens. The relatively lower performance in the Maurer-Stroh dataset can be due to this dataset being especially challenging, as it contains allergens and (potential) non-allergens with the same structural folds (Maurer-Stroh *et al.* 2019). Particularly encouraging is the high performance (AUC close to 0.9) in Sharma’s dataset, which is very large (1 order of magnitude larger than our training set) and also includes non-plant allergens (Fig. 5).

The base-line strategy for predicting protein allergenicity (i.e. IgE cross-reactivity), and that established by the Codex Alimentarius Commission (World Health Organization, Food and Agriculture Organization of the United Nations 2009, Codex Alimentarius Commission 2003), is based on sequence similarity against other known allergens. In our tests, that approach also yields a good performance. This outcome reflects the fact that most plant food allergens in this dataset belong to well-characterized protein families with close homologs already present in allergen databases, conditions under which BLAST-based screening is expected to perform well. Our predictor, apart from the higher global accuracy, outperforms sequence similarity in terms of sensitivity, hence offering a more flexible and generalizable framework. Sensitivity is particularly important in allergenicity prediction, as failing to identify true allergens (false negatives) can have serious safety implications. While over-prediction (false positives) can lead to additional confirmatory testing *in vitro* or *in vivo*. Both sensitivity and specificity are important, and a balanced predictive approach is needed to minimize false negatives without generating excessive false positives.

Analyzing the false positives and negatives of the *k*-fold evaluation, we find some interesting features. Among the few false negatives (allergens not detected as such), most them belonged to under-represented families (e.g. defensins, gibberellin-regulated proteins, thioredoxins, pollen polcalcins; Ara h 13, Cit s 7, Tri a 25, Bra r 5) and/or were clinically relevant via inhalation or occupational exposure rather than ingestion (e.g. Tri a 41, Tri a 43, Tri a 45, Bra r 5). It is also worth mentioning that many of these false negatives had a low prediction score, suggesting that applying a more restrictive cutoff could reduce them, albeit at the expenses of including more false positives. Regarding the false positives (proteins incorrectly classified as allergens), many arise from structural or functional convergence with known allergens, as small, secreted, cysteine-rich defense peptides that share features such as high cysteine content, compact disulfide-stabilized folds, and protease resistance, leading embeddings to place them near canonical allergens. Other false positives stem from short or fragmentary sequences, including proteomics-derived peptides, which lack contextual information and can be misinterpreted as immunogenic motifs. Database quality issues, such as unreviewed, flagged, or soon-to-be-removed entries, also contribute by introducing noisy or artifactual sequences. In addition, many high-scoring false positives are proteins involved in plant defense. This reflects a functional bias in the model as it may associate defense-related features with allergenicity, even if there is no clinical evidence that these proteins actually cause allergic reactions. Finally, remote homology or domain-level confounding can cause occasional false positives in proteins containing motifs superficially similar to allergens, despite lacking canonical secretory or exposure characteristics.

The strong performance of the classical similarity-based approach in our benchmark should be interpreted in the context of dataset composition. Because the present dataset focuses on plant food allergens, most entries have close sequence relatives among well-studied allergen families such as lipid-transfer proteins, storage globulins, or pathogenesis-related proteins. Under these conditions, the WHO/FAO similarity criterion remains highly effective, as it directly captures cross-reactivity driven by sequence homology. However, such performance is unlikely to generalize to broader protein spaces, where novel or divergent sequences dominate and explicit homologs are absent. In contrast, the embedding-based model encodes biochemical and contextual information that extends beyond direct similarity, providing a foundation for detecting potential allergens from underrepresented families or new food sources. Thus, both approaches can be viewed as complementary: similarity-based searches excel in domains with well-known allergen families, while embedding-based predictors offer broader coverage and mechanistic interpretability.

The input for this predictor is raw protein amino-acid sequences, and hence it can be readily applied to the sequenced genomes of new species planned to be incorporated as food sources. Similarly, the predictor is easily updatable and can be re-trained as databases grow in the future incorporating new experimentally tested allergens. The representation of these sequences as vectorial embeddings ensures that many latent structural features are implicitly considered, as these embeddings are known to encode important information about the structure and function of the proteins (Rives *et al.* 2021). This representation of protein sequences is being used for many different purposes in protein bioinformatics, and recently, allergenicity predictors also based on sequence embeddings have started to be developed, although none of them is focused on plant proteins. For example AllerTrans combine two different vector embeddings as input for a neural network trained to discriminate allergens and non-allergens (Sarlakifar *et al*. 2025). PreAlgPro also uses ProtT5 embeddings as input for a neural net classifier (Zhang and Liu 2024). While these systems use embeddings extracted from the primary sequences, it cannot exclude that incorporating explicit structural information could further improve predictive performance.

Overall, our findings confirm that homology-based screening remains an effective baseline for established allergen families, especially in narrowly defined datasets such as plant food allergens. Yet, embedding-based representations offer a scalable and more general solution for emerging proteins where sequence similarity offers limited guidance. As genome sequencing expands to new crops and novel food sources, predictors that capture latent structural and functional signals, rather than explicit homology, will be increasingly valuable. Future work should therefore integrate both paradigms in a tiered, weight-of-evidence pipeline that combines conservative similarity filters with embedding-based scoring, exposure-route annotation, and experimental validation.

## Supplementary Material

vbag114_Supplementary_Data

## Data Availability

The predictor is open-source and freely available at GitHub (https://github.com/Martinmendezlopez/ALLERGEN-PREDICTOR-FROM-ProtTrans/).
