# Supplementary material for: Prediction of plant food allergens using protein embeddings
Source: Bioinform Adv. 2026 Apr 26;6(1):vbag114. doi: 10.1093/bioadv/vbag114 (PMC13162349; doi:10.1093/bioadv/vbag114)
Supplement: vbag114_Supplementary_Data [file vbag114_supplementary_data.pdf]

Prediction of plant food allergens using protein embeddings

Supplementary Material

Martín Mendez[a], F. Javier Moreno[b] Florencio Pazos[a*]

[a] Computational Systems Biology Group, National Centre for Biotechnology (CNB-CSIC), 28049 Madrid, Spain.

[b] Instituto de Investigación en Ciencias de la Alimentación (CIAL), CSIC-UAM, CEI (UAM+CSIC), 28049 Madrid, Spain.

* Correspondence to: pazos@cnb.csic.es

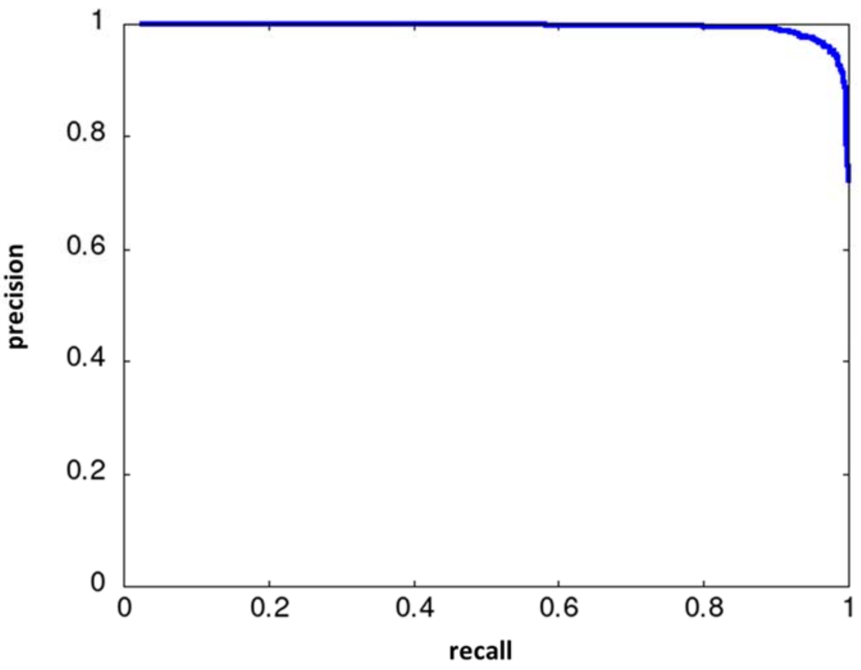

Figure S1. Precision/recall curve illustrating the discriminative power of the method in the plant food allergens/non-allergens dataset.

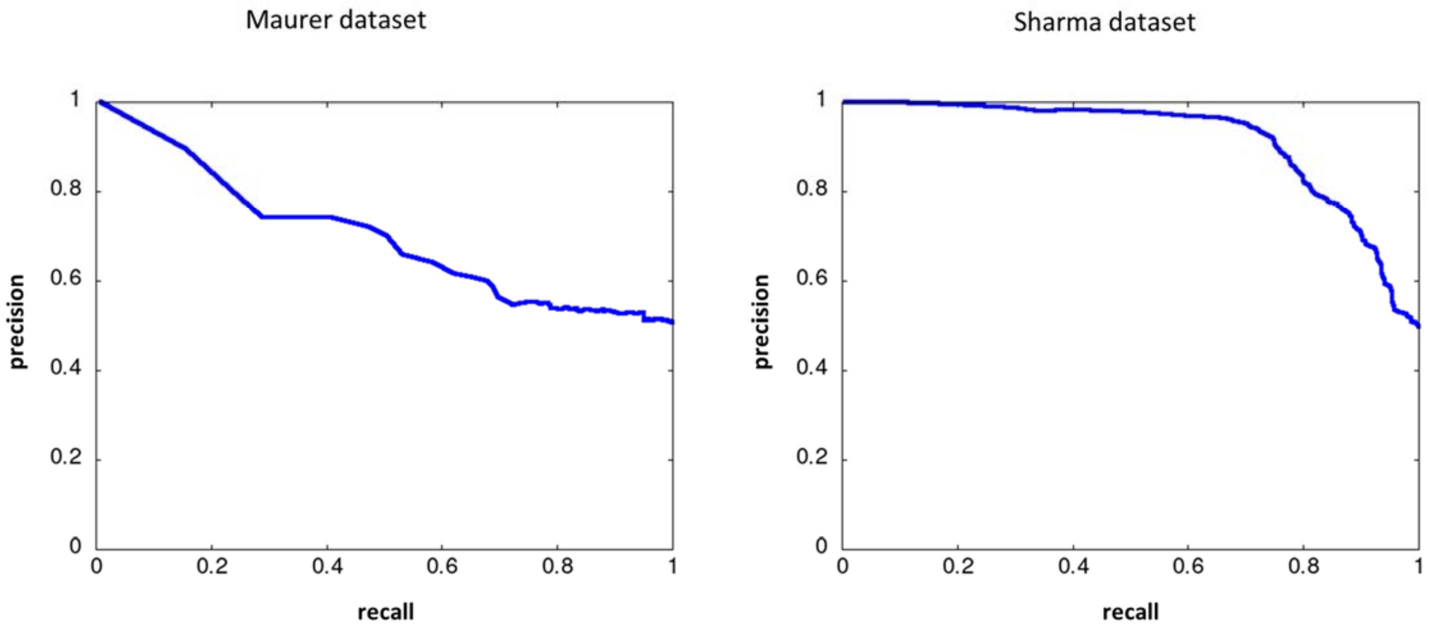

Figure S2. Precision/recall curves for Maurer-Stroh et al. and Sharma et al. datasets.
